# The Impacts of Perceived Risk and Negative Emotions on the Service Recovery Effect for Online Travel Agencies: The Moderating Role of Corporate Reputation

**DOI:** 10.3389/fpsyg.2021.685351

**Published:** 2021-05-31

**Authors:** Jiahua Wei

**Affiliations:** Business School, Guilin University of Technology, Guilin, China

**Keywords:** OTA, service recovery effect, perceived risk, negative emotions, corporate reputation

## Abstract

This study explores the impact mechanism of perceived risk and negative emotions on the service recovery effect of an online travel agency (OTA) through a scenario experiment. The results show that: perceived risk has positive and negative impacts on negative emotions and service recovery satisfaction, negative emotions have a negative impact on service recovery satisfaction, and corporate reputation plays a positive moderating role in the relationship between perceived risk and service recovery satisfaction. This study is helpful to better explain the impact mechanism of the service recovery effect of OTAs, and to provide a theoretical reference for improving the service recovery effect of OTAs.

## Introduction

In the Internet age, the huge demand from consumers for online travel has spawned online travel agencies (OTAs) that resemble a kind of consumption fashion (Pinto and Castro, 2019). The OTA Expedia was founded in 1996 (Ling et al., 2014), and it has become the online sales channel with the highest booking rate. The main function of the OTA is to obtain commission by providing travel related services, integrate products and reduce costs, and provide consumers with cheaper solutions (Kim et al., 2009). In essence, OTAs are intermediaries that rely on Internet platforms to provide online consulting, commenting, and booking services; because OTAs can easily compare products, prices, discounts, independent reviews, and photos of various hotels to meet the needs and preferences of consumers, they have developed rapidly, and some famous brands have appeared, such as Priceline, Expedia, and Ctrip.

OTAs have been developing rapidly globally, but they are facing the challenge of service failure. At present, OTA service failure events reported by the media are endless. For example, the “10.1” holiday in 2020 is the first holiday in China since its COVID-19 situation went under control. Some Chinese OTA brands, such as Ctrip, Qunar, and Tuniu, have become the object of consumer complaints. Mr. Han of Hangzhou, China, is a tourist who experienced failure in the OTA service. On October 1, 2020, he booked a reservation at a hotel in Sanya, China through the online platform of Ctrip, a famous OTA in China. On October 2, 2020, Mr. Han and his family arrived at the hotel and found that the hotel room was not as large as Ctrip had promised. The sanitation was poor, and it was not convenient to wash in the hotel room. Therefore, Mr. Han asked for a refund, but the hotel and Ctrip refused to give a full one, only 20%, and members of the hotel service staff were arrogant. In the dispute, they attacked Mr. Han personally, causing his anger. Later, Mr. Han made a complaint through the Chinese consumer protection agency and exposed it through the media, which had a negative impact on the image of Ctrip and the hotel.

From the OTA case of Mr. Han, it can be seen that service failure has become a lingering curse for OTAs and service providers (such as hotels), which has created new challenges for the OTA service operation, so service recovery is urgently needed. Service failure is a kind of service that fails to meet customer expectations and that causes customer dissatisfaction (Peng and Jing, 2005; Nikbin et al., 2015). The current literature discusses the quality measurement system of online shopping service recovery (Liu and Li, 2017) and the relationship between online shopping service, service recovery, and customer satisfaction and loyalty (Jian and Ke, 2017). At present, academic circles have also studied the impact of word-of-mouth on the service recovery effect of travel agencies, and the service recovery effect is measured by customer satisfaction and customer loyalty (Pai et al., 2019). They developed a practical manual on service failure and service recovery for the travel industry (Inkson, 2019). However, the current literature still lacks research results on OTA service failure and service recovery, so in reality it cannot fully explain and guide the service failure and service recovery of OTAs, thus it needs to be further studied.

Perceived risk is a derivative concept of psychological research, and it refers to the uncertainty and risk perceived by consumers in the process of purchasing products or services (Jeon et al., 2020). The impact of perceived risk on negative emotions has been discussed in academic circles, such as in the studies conducted by Yoon and Lee (2014), Mi et al. (2019), and Zheng et al. (2019). It is generally believed that perceived risk will strengthen the negative emotions of customers. However, the current research still lacks relevant research results in the context of OTA service failure and service recovery. Corporate reputation is also operational. It can affect customer perceived value and then affect consumer intention and decision-making behavior (He et al., 2018). An OTA is a kind of intermediary that provides online consultation, comment, and reservation services based on an Internet platform, and it is also an online platform. With the rise in the number of Internet services, some researchers, such as Li (2014), Zhao and Wang (2012), and Olavarria-Jaraba et al. (2018), have studied the reputation of online platforms. In the service recovery scenario, whether the corporate reputation of OTAs will play a moderating role among some variables still lacks discussion.

The study uses a scenario experiment to conduct empirical analysis, and innovatively introduces perceived risk, negative emotions, and corporate reputation into the research on the impact mechanism of the service recovery effect of OTAs. Specifically, this study will explore the relationship between perceived risk and service recovery satisfaction, negative emotions and service recovery satisfaction, and service recovery satisfaction and customer loyalty, and verify the moderating role of corporate reputation between perceived risk and service recovery satisfaction. The study helps to expand the vision of service recovery research of OTAs, better explain the impact mechanism of service recovery effect of OTAs, improve the service recovery effect of OTAs, promote the healthy development of OTA business models, and bring more value to society and customers.

## Literature Review and Research Hypothesis

### Online Travel Agency

Rianthong et al. (2016) have suggested that the development of Internet technology has profoundly changed the way tourists book appointments, and online booking channels have developed greatly, including OTAs. The first OTA, Expedia, was established in 1996 (Ling et al., 2014). Since then, OTAs have developed rapidly all over the world. OTAs provide an effective platform for consumers to browse, purchase travel services, and share travel information through websites, mobile devices, apps, and call centers. An OTA is a tool for marketing, searching, and booking; it can earn tourism market share by managing hotel room reservations (Sheng, 2019). In essence, the OTAs are intermediaries that rely on an Internet platform to provide online consultation, comment, and reservation services. In the hotel and tourism industries, OTAs, through online distribution channels, play a key role in providing consumers with more attractive products (Kim and Lee, 2004). OTAs have profoundly changed the way consumers buy tourism products, and they have a great development potential. Traditional tourism enterprises have also strengthened cooperation with them to meet the needs of tourists (Sheng, 2019).

The OTAs help to increase the visibility of a hotel, thus increasing the interest and occupancy rate of tourists (Ling et al., 2015). OTAs have played an important role in building the reputation of a hotel. One of the factors contributing to the success of Weiganghui Hotel in Hong Kong was the close cooperation with OTAs at the beginning of its operations and the expansion of its promotion scope (Tony, 2013). However, some scholars have different views on this issue, believing that OTAs have little value but they have gained a lot of income that should belong to hotels (Green and Lomanno, 2012). For consumers, booking travel services through OTAs has several advantages: easy to use, low price, time-saving, comfortable, and diversified service products (Liu and Zhang, 2014; Hao et al., 2015; Pappas, 2017). However, there is still a lack of discussion on OTA service failure and service recovery, and it is urgent for the academic community to conduct in-depth research on this topic in order to promote the healthy development of OTA business models.

### Service Failure and Service Recovery

Service failure refers to the when the service provided by a service enterprise fails to meet the minimum acceptable standard of customers, and fails to meet the requirements and expectations of customers, resulting in customer dissatisfaction (Chaouali et al., 2020). From the perspective of customer expectations, service failure is a kind of service that fails to meet customer expectations and a contact environment that causes customer dissatisfaction (Peng and Jing, 2005; Nikbin et al., 2015). Since the 1980s, service failure has attracted the attention of scholars. Scholars have conducted extensive discussions on the definition, classification, causes, influencing factors, and consequences of service failure, enriching the research on service failure (Bitner et al., 1990; Maxham, 2001; Sven et al., 2015; Liu et al., 2019).

After the service failure of an enterprise, to repair its image and recover loss, service recovery is urgently needed. Service recovery is not only an action taken against service failure, it is also the hard work of an enterprise to make the service meet the expectation of a customer (Zhong et al., 2011). Some scholars have discussed service recovery strategy, and they think that it is necessary to confirm, evaluate, explain, apologize, and compensate for service failure, so as to improve the satisfaction of service recovery (Boshof, 1999). Heejung (2012) has suggested that service recovery should consider personality and preference, such as taking different service recovery strategies according to the education background, age, and preference of a customer. The influence mechanism of service recovery satisfaction is a research hotspot. The current literature focuses on the influence of customer misconduct, time perception, cultural differences, customer psychological contract violation, economic compensation, and emotional compensation on customer satisfaction after service recovery (Valenzuela and Cooksey, 2014; Albrecht et al., 2017).

Generally speaking, the current research on service failure and service recovery is relatively active, which lays a theoretical foundation for this study. However, the current literature still lacks OTA service failure and service recovery research results, so there is a need to continue in-depth study.

### Perceived Risk and Negative Emotions

Perceived risk is a derivative concept of psychological research, and refers to the uncertainty and risks perceived by consumers in the process of purchasing products or services (Jeon et al., 2020). Perceived risk is subjective. No matter how big the actual risk of a product or service is, if it is not perceived by consumers, it will not affect their consumption behavior (Wang et al., 2018). Cui (2015) believes that perceived risk exists in six aspects: body, performance, property, time, society, and psychology. Sjöberg (2007) found that emotion does play an important role in perceived risk and related attitude, in which people found that interest in risk (positive emotion) is positively correlated with perceived risk. In the online shopping situation, because of the virtual characteristics of a network environment, the online shopping behavior of consumers often faces more perceived risks. Some scholars divide perceived risk into information, transaction, distribution, and after-sales (Wang, 2020), while others divide perceived risk into financial, functional, time, and privacy (Ren et al., 2019). For the research on influencing factors of perceived risk, Byun and Ha (2016) discussed the relationship between information usefulness, source credibility, perceived risk, impulsive purchase, and purchase intention in online shopping behavior. The research shows that information usefulness has a negative impact on perceived risk. Ren et al. (2019) suggested that in an online shopping scenario, the reputation, product quality, and website construction of a seller and other factors significantly negatively affect the financial, functional, and time risks in perceived risk, and that logistics support significantly negatively affects the financial, functional, time, and privacy risks in perceived risk.

Emotion is a psychological concept, and refers to the response of an individual to a specific object with external stimulation. Customer emotion is the synthesis of a series of related emotional reactions generated in the consumption process (Jia and Zhao, 2018). Previously, Westbrook and Oliver (1991) proposed that customer emotion can be divided into five dimensions: pleasant surprise, unpleasant surprise, anger, happiness, and sadness or indifference. However, most scholars divide emotions into positive and negative. Positive emotions include state of high energy activation, concentration, happiness, and engagement; negative emotions include anger, complaint, depression, regret, and helplessness (Berry et al., 2010; Jaeger et al., 2018). Liljander and Strandvik (1997) have suggested that customers will experience different positive and negative emotions on service, which affects customer satisfaction. Among them, negative emotions have a great impact on customer satisfaction. Ou and Verhoef (2017) have explored the incremental effects of positive and negative emotions on loyalty intention. The effects of these two emotions on loyalty intention are increasing, and positive emotions weaken positive connection (negative interaction). Khatoon and Rehman (2021) have stated that the negative emotions of consumers about a brand can be directly translated into actions against it, such as spreading negative word-of-mouth, avoiding, and retaliating.

The impact of perceived risk on negative emotions has been discussed in the current academic community. Zheng et al. (2019) conducted an empirical study on the Wenchuan earthquake-stricken areas in China and found that the perceived risk of residents in the disaster areas will enhance negative emotions. Some researchers have explored the impact of risk perception on subsequent behavior of apartment hotels. Studies show that perceived risk of apartment hotels has significant positive and negative effects on negative emotions and trust (Mi et al., 2019). Lee and Jung (2015) discussed the relationship between perceived risk and negative emotion in online shopping. The research shows that the perceived risk of customers will enhance their negative emotions. Yoon and Lee (2014) conducted an on-the-spot survey on tourists at the Seoul Lantern Festival. The results show that perceived risk has a positive impact on negative emotions. When the OTA service failure, such as being threatened by service personnel or unable to procure a refund occurs, customers will lose their spirit and money, and the perceived risk of customer dissatisfaction will arise. This will enhance the negative emotions of customers, including anger, complaint, depression, regret, and helplessness. Therefore, the study proposes the following hypothesis:

H1: Perceived risk has a significant positive impact on negative emotions.

### Service Recovery Satisfaction

Since the 1960s, customer satisfaction has been a hot issue in service research. At present, there are two main perspectives to define customer satisfaction. The specific perspective of customer satisfaction is that customer satisfaction is a kind of immediate emotional reflection of customer satisfaction with the value obtained after consumption in a specific situation; while customer satisfaction from an overall perspective is that customer satisfaction is a holistic attitude based on experience formed by customer attitude (Woodside et al., 1989). Combined with its characteristics, the study will adopt a specific perspective to define customer satisfaction. It is considered that service recovery satisfaction is a kind of feeling state in which the actual perceived effect of instant service recovery is higher than the expected service recovery. Its most prominent feature is a kind of customer satisfaction in a specific situation of service recovery, which is a kind of “second-degree satisfaction.”

The relationship between perceived risk, customer satisfaction, and service recovery satisfaction has been studied in the academic field. Wang et al. (2017) took customers in the mobile communication industry of China as research samples, and the research confirmed that customer perceived risk would reduce customer satisfaction, and that customer participation would reduce customer risk perception. Yao and Deng (2017) suggested that the two dimensions of perceived risk include financial and time risks, and that these risks have a negative impact on customer satisfaction. Hsieh and Tsao (2014) pointed out that in a network environment, user perceived risk has a significant negative impact on user satisfaction, and that the higher the perceived risk, the lower the user satisfaction. Scridon et al. (2020) conducted an empirical study on the Romanian market, and the results showed that the perceived risk of customers has a significant negative impact on customer satisfaction and customer loyalty, and ultimately affects the profits of enterprises. Chang and Hsiao (2008) conducted an empirical study on the service recovery of hotels, and suggested that perceived risk will reduce customer value and that the decrease in customer value will inevitably reduce customer satisfaction. However, there is no research on the relationship between perceived risk and customer satisfaction in an OTA service recovery situation. In the OTA service recovery scenario, if the perceived risk of customers increases, the customer satisfaction reduces, and if the perceived risk of customers is reduced, the customer satisfaction is improved. Therefore, the following hypothesis is put forward:

H2: Perceived risk has a significant negative impact on service recovery satisfaction.

Westbrook (1991) studied the relationship between emotions and satisfaction. It is suggested that the positive emotions of customers have a positive effect on satisfaction, and that negative emotions have a negative effect on satisfaction. Yoon and Lee (2014) conducted an on-the-spot investigation on the tourists at the Lantern Festival in Seoul, South Korea, and analyzed them using a structural equation model. The results showed that perceived risk has a positive impact on negative emotions, and that negative emotions will reduce customer satisfaction. In the context of service recovery, Du and Fan (2007) explored the relationship between negative emotions and service recovery satisfaction using the method of simulation experiment. The results showed that the negative emotions of customers were negatively correlated with the service recovery satisfaction. In the OTA service recovery scenario, when the negative emotions of customers increase, the satisfaction of service recovery decreases. Therefore, the following hypothesis is put forward:

H3: Negative emotions have a significant negative impact on service recovery satisfaction.

### Corporate Reputation

Reputation plays an important role in business. A good reputation can bring higher reputation benefits to enterprises, attract better employees, reduce costs, gain price advantages, and reduce enterprise risks (Dong, 2015). Corporate reputation also has operability. It can affect customer perceived value and then affect consumer intention and decision-making behavior, and it can help enterprises transform or obtain favorable factors from other aspects (He et al., 2018). With the rise of Internet services, some researchers have studied the reputation of online platforms. Olavarria-Jaraba et al. (2018) think that because of the lack of sound network supervision and social credit systems, if the default of a borrower is not curbed in time, the reputation risk of an online platform will increase. A good reputation is not only the precondition for P2P to charge higher service fees but also an effective incentive to improve the service level of fund lending and establish and maintain the reputation of an online platform (He et al., 2018; Olavarria-Jaraba et al., 2018).

In essence, an OTA is an intermediary organization that provides online consultation, comment, and reservation services based on an Internet platform, and it is also an online platform. Although the current literature on the impact mechanism of online platform service recovery effect is still very lacking, there is also a small amount of related research. Some studies suggest that online platform enterprises can become a collective signal of online platform reputation through social discussion of electronic word-of-mouth, thus driving customer demand, and that the establishment of online platform reputation can maintain long-term psychological contract relationship and reputation (Li, 2014). The credibility of online platform reputation information perceived by online platform buyers has a positive impact on the use level of reputation information and online shopping amount (Zhao and Wang, 2012). In the OTA service recovery scenario, if the OTA has a good corporate reputation, it will increase the sense of trust and satisfaction of a customer, which is helpful in enhancing service recovery. Therefore, the following hypothesis is put forward:

H4: Corporate reputation plays a positive moderating role in the relationship between perceived risk and service recovery satisfaction.

### Customer Loyalty

Customer loyalty is the deep commitment of customers to their preferred enterprises or brands from which they will continue to buy repeatedly in the future. No matter how the situation or marketing power influences, customer loyalty will not produce switching behavior (Oliver, 1999). Loyal customers are the source of competitive advantage and an important guarantee of enterprise development. In the dimension research, customer loyalty can be divided into cognitive component, emotional component and behavior, reconstruction intention, repeat purchase, recommendation to others, and attention (Li, 2014). In the online shopping environment, the academic research combines traditional customer loyalty driving factors and online shopping environment to study customer loyalty in online shopping. Luarn and Lin (2003) studied the driving factors of online shopping loyalty and found that customer trust, customer satisfaction, and customer perceived value have a positive impact on customer loyalty. Yu et al. (2008) took the traditional service industry as the research object, selected customer samples from Changsha, Hangzhou, Guangzhou, and seven other cities in China for empirical research, and found that service recovery satisfaction has a significant positive impact on customer loyalty. In the case of OTA service recovery, when customers are satisfied with OTA service recovery, they will inevitably produce behavioral loyalty and attitudinal loyalty. Therefore, the following hypothesis is put forward:

H5: Service recovery satisfaction has a significant positive impact on customer loyalty.

From the above analysis and hypothetical relationship, we can see that in the OTA service recovery scenario, negative emotions play a mediating effect between perceived risk and service recovery satisfaction. At the same time, perceived risk has an impact on customer loyalty through two variables: negative emotion and service recovery satisfaction. Negative emotion and service recovery satisfaction play a mediating effect between perceived risk and customer loyalty. Therefore, this study proposes two hypotheses for mediating effects:

H6: Service recovery satisfaction plays a mediating effect between perceived risk and customer loyalty.
H7: Negative emotions and service recovery satisfaction play a mediating effect between perceived risk and customer loyalty.

**Figure 1:**
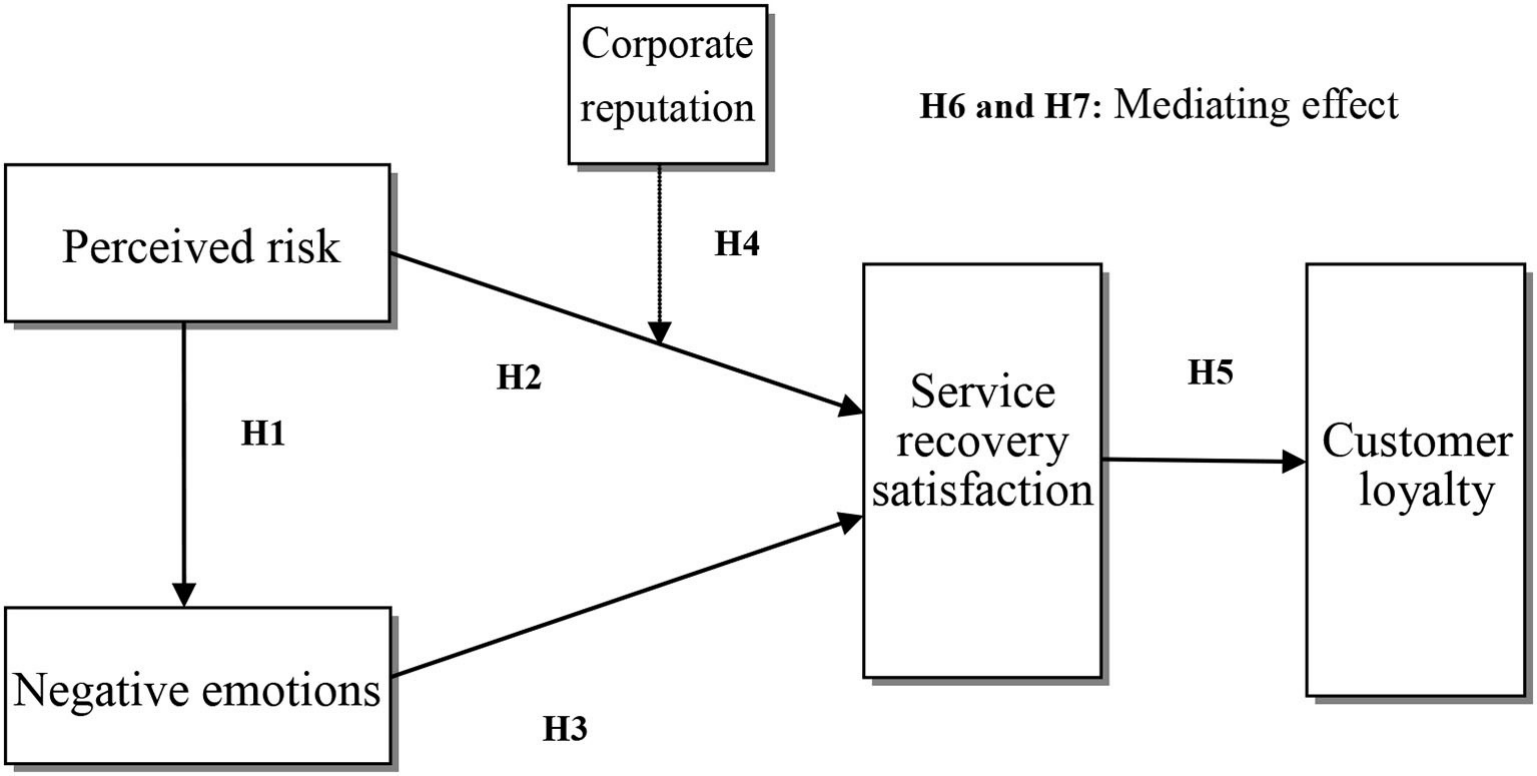
Research model.

Based on the above seven hypotheses, a research model is proposed, as shown in Figure 1.

## Research Design

### Experimental Design

The purpose of the study is to explore the influence mechanism of perceived risk and negative emotions on service recovery effect after OTA service failure. If the questionnaire survey method is used, only the respondents can be asked to recall the service failure and service recovery experience to fill in the relevant content, and the results may have a large memory bias (recall bias), affecting the accuracy of the research results. In contrast, the experimental method based on scenario description has higher internal validity. Combined with the characteristics of this study, based on the experimental research methods of Du and Fan (2007) and Poggi and D'Errico (2010), we decided to use the scenario experiment method for empirical research.

In order to improve the external validity of the situational experiment, the materials of the situational experiment are obtained from the OTA service failure events reported by the Chinese media. At the same time, this study has also referred to the online tourism complaints published by the China National Tourism Administration, and has made an appropriate adaptation to make it more suitable for scenario experimental research.

The service recovery scenario is designed as 2 (high perceived risk vs. low perceived risk) × 2 (high negative emotions vs. low negative emotions) × 2 (high corporate reputation vs. low corporate reputation). To avoid the mixed effect caused by the existing relationship between customers and real OTAs, the OTA brand, hotel name, and travel route name in the experimental situation are fictitious. The experiment was divided into three parts.

The first part describes the perceived risk of customers. (1) Under the experimental conditions of high perceived risk, the study describes that the location of the hotel where the OTA arranges customers to stay is not good, and that the room facilities and sanitary conditions are also relatively poor, which is inconsistent with the introduction on the OTA platform. Customers ask for a refund, but the OTA and the hotel refuse to refund. In negotiation, the service attitude of the hotel service staff was also poor, and they attacked the customers with language. Subsequently, the customers complained to the OTA and the hotel. (2) Under the experimental conditions of low perceived risk, this paper describes that the OTA arranges customers to stay in a hotel. The room facilities of the hotel are basically consistent with the introduction on the OTA platform, but the customers are not very satisfied with the hotel location and sanitation, and they think the price is relatively high. The customers have not complained about the OTA.

The second part describes the negative emotions of customers. (1) Under the experimental conditions of high negative emotions, the hotel location, room facilities, and sanitary conditions arranged by the OTA were poor, and the service attitude of the service staff was also poor, and the staff even made verbal attacks on customers. The customers feel very angry, frustrated, regretful, and helpless, and the negative emotions are very obvious. (2) Under the experimental condition of low negative emotion, this study describes that after the OTA arranges customers to stay in the hotel, although the room facilities are basically consistent with the introduction on the OTA platform, the customers are not satisfied with the hotel location, room hygiene, and other conditions. On the whole, the degree of negative emotions of the customers is not so high, just slight negative emotions, such as slight complaints and regrets about service details, which are attributed to bad luck. The customers did not show serious anger, depression, and other negative emotions.

The third part describes the corporate reputation of an OTA. (1) Under the experimental conditions of high corporate reputation, the OTA enjoys a high level of reputation, and its service quality has a good reputation, which is welcomed by customers and the public. (2) Under the experimental condition of low corporate reputation, the study describes that the OTA has low popularity and reputation, and that its word-of-mouth is also relatively poor. There are frequent reports of OTA service failure in the media, and it is not welcomed by customers and the public.

Since in the scenario experiment the service recovery scenario is designed as 2 (high perceived risk vs. low perceived risk) × 2 (high negative emotions vs. low negative emotions) × 2 (high corporate reputation vs. low corporate reputation), there are eight scenarios in total. The researcher asked the subjects to put themselves in a certain situation, explained various scenarios, and guided the subjects to imagine their feelings and reactions as OTA customers in the service failure and service recovery scenarios of the OTA. Then, the researcher instructed the subjects to fill in the questionnaire of the situational experiment.

### Variable Measurement

The measurement variables include perceived risk, negative emotions, corporate reputation, service recovery satisfaction, and customer loyalty. In order to ensure the pertinence and effectiveness of measurement of the five variables, this study adopts the relevant scales of authoritative literature and makes corresponding modifications according to the OTA service recovery scenarios.

In the study, the measurement of perceived risk draws on the scales of Li and Li (2007) and Lee and Moon (2015), and eight items are set. In the development of the scale of negative emotions, seven Chinese residents with OTA service failure and recovery experience were invited to speak about the related negative emotions in words in the OTAs service recovery situation, and then the frequency of these words was calculated and sorted to form the initial negative emotion scale. On this basis, the study referred to the emotion measurement scale used by Baron et al. (2005) and Fang et al. (2019), and finally set five items for negative emotion variables. Based on the research results of Ponzi et al. (2011), and Wang and Guo (2018), the corporate reputation scale has five items. According to Bhatta and Premkumar (2004) and Ribbink et al. (2004), the measurement of service recovery satisfaction is modified according to the characteristics of OTA service failure and service recovery. The measurement of customer loyalty is based on the customer loyalty scale compiled by Zeithaml et al. (1996), and four items are set up, including two aspects of customer behavior loyalty and customer attitude loyalty. On this basis, the study also invited 15 Chinese citizens with the OTA service experience, and four psychology and management researchers to revise the text content and expression of the first draft of the questionnaire, and correct the ambiguous meaning and unclear expression in the questionnaire. All of the items were measured using a five-point Likert scale with the options “very consistent,” “consistent,” “basically consistent,” “not very consistent,” and “not consistent.”

### Pre-experiment

Before the implementation of the formal experiment, to ensure the reliability of the experimental situation and the effectiveness of experimental manipulation, the researchers conducted a pre-experiment on college students. The college students participated in the pre-experiment as volunteers. The reason why the pre-experiment chooses the college students as experimental samples is that the internal consistency of the college students is high, and the college students are curious and easy to accept new things. They are the active group of OTA consumption, and they have a better understanding of the OTA operation and consumption process, which has a good experimental value.

Eighty-two college students, 39 females (47.6%) and 43 males (52.4%), who had OTA consumption experience participated in the pre-experiment. Independent sample *T*-test was conducted on the questionnaire data. The test results show that the credibility of the experimental scenario is relatively high (the average is 3.83), and that the evaluation of perceived risk, negative emotions, and corporate reputation of the subjects in different experimental scenarios is consistent with the experimental design. Under the conditions of high perceived risk, the perceived risk of OTA service failure was higher (M _high perceived risk_ = 4.46, M _low perceived risk_ = 2.97; *T* = 2.943, *DF* = 54, *P* < 0.01). Under the experimental conditions of high negative emotion, the negative emotion of OTA service failure was stronger (M _high negative emotions_ = 4.48, M _low negative emotions_ = 3.29, *T* = 2.819, *DF* = 73, *P* < 0.01). Under the experimental conditions of high corporate reputation, the subjects rated the corporate reputation of the OTA better (M _high corporate reputation_ = 4.62, M _low corporate reputation_ = 3.37, *T* = 2.311, *DF* = 62, *P* < 0.05). The independent sample *T*-test results of the above pre-experiment show that the variables of perceived risk, negative emotions, and corporate reputation are manipulated successfully.

### Formal Experiment

From October to November 2020, the study conducted a formal experiment. The experimental samples were from Guilin, Nanning, and Liuzhou, and farmers from the Yangshuo and Luzhai counties in China. The formal experiment was composed of researchers, four university volunteers, and 12 basic cadres of communities and villages. The subjects were recruited in the formal experiment through the following two ways. The first was through cooperation with the communities in Guilin, Nanning, and Liuzhou (community is grassroots management organizations in Chinese city) to collect experimental samples through social platforms (QQ group and WeChat group). The subjects were required to have OTA service purchase experience. The second was through cooperation with the villages of the Yangshuo and Luzhai counties (village is grassroots management organization in Chinese rural areas) and collection of subjects through the social platform (QQ group). The subjects were also required to have OTA service purchase experience.

Because the subjects lived in different areas, the formal experiment was completed 11 times. The service recovery scenario designed in this study is 2 (high perceived risk vs. low perceived risk) × 2 (high negative emotions vs. low negative emotions) × 2 (high corporate reputation vs. low corporate reputation). In each experiment, the researcher explained various situations, asked the subjects to choose one of the scenarios, read the text description of their own situation carefully, and put the individual under the situation. The researcher guided the subjects to imagine their feelings and reactions as OTA customers in a certain situation of OTA service failure and service recovery. Then, the researcher instructed the subjects to fill in the questionnaire of the situational experiment. To improve the quality of the questionnaire, the researchers provided necessary guidance. At the same time, in order to ensure the filling effect and respect personal privacy, the subjects were asked to complete the answers in free private space.

Formal experiments focus on solving the problem of social desirability bias. Scholars believe that there are three main reasons for the occurrence of social desirability bias: the subjects themselves have higher social desirability, the test situations stimulate the social desirability bias of the subjects, and the items themselves cause social desirability bias (Paulhus et al., 2002). In this experiment, the subjects involved ordinary Chinese citizens and farmers who did not have high social desirability and would make a more objective evaluation based on facts. There is no social desirability in the guidance of the questionnaire. We have also optimized the expression of items to prevent the occurrence of social desirability bias. Before the test, we told the subjects that the questionnaire was anonymous and would not reveal privacy, so that they could fill in the questionnaire objectively and safely.

In the process of investigation, there are many factors that lead to the missing or non-response of some individuals in the sample, so the final valid sample is only a part of the survey sample (Smironva et al., 2019). Therefore, formal experiments also focus on solving the problem of non-response bias. First of all, when the subjects are recruited through community and village committees, they are required to choose to participate in the experiment voluntarily and have the OTAs service purchase experience, which greatly reduces non-response bias. Second, in the formal experiment, we trained the subjects and informed them of the experimental operation process and questionnaire filling and answering methods, which improved the accuracy of the questionnaire and avoided the question of non-response bias.

A total of 388 questionnaires were collected in the formal experiment, and 355 questionnaires were valid. The effective rate was 91.26%. The sample distribution is shown in Table 1.

**Table 1:** Sample distribution.

**One-Class indicators**	**Two-Class indicators**	**Sample size**	**Percentage**	**One-Class indicators**	**Two-Class indicators**	**Sample size**	**Percentage**
Gender	Male	172	48.45%	Education	Secondary school and below	121	34.08%
	Female	183	51.55%		College degree	123	34.65%
Region	Guilin city	104	29.30%		Bachelor	78	21.97%
	Nanning city	86	24.23%		Master and doctor	32	9.01%
	Liuzhou city	77	21.7%	Occupation	Enterprise staff	74	20.85%
	Yangshuo county	47	13.24%		Professional	61	17.18%
	Luzhai county	41	11.55%		Self-employed person	54	15.21%
Age	Under 25 years	95	26.76%		Civil service staff	23	6.48%
	26–35 years	103	29.01%		Student	42	11.83%
	36–59 years	127	35.77%		Farmer	68	19.15%
	Over 60 years	30	8.45%		Other	33	9.3%

## Data Analysis

### Reliability and Validity Test

Cronbach's α is an index used to measure the reliability of the questionnaire data. Cronbach's α is required to be greater than the threshold value of 0.7. The higher the Cronbach's α is, the stronger the internal consistency of the questionnaire is and the higher the reliability is (Nunnally and Bemstein, 1994; Hair and Black, 2006). As shown in Table 2, the Cronbach's α of the five variables ranged from 0.741 to 0.882. If the Cronbach's α is >0.7, it means that reliability has passed the test (Hair and Black, 2006), indicating that the internal consistency of the questionnaire met the requirements, and that the reliability of the questionnaire passed the test.

**Table 2:** Test for reliability and convergent validity.

**Variables**	**Items**	**Normalized load factor**	***T*-value**	**Cronbach's α**	**CR**	**AVE**
Perceived risk	1. The OTA may have forged its credit record	0.783	3.481	0.882	0.921	0.597
	2. The complaint was not handled in time	0.761	4.827			
	3. My payment cannot be returned	0.691	2.959			
	4. Poor attitude of service staff	0.903	4.724			
	5. I am worried about wasting a lot of time	0.731	3.765			
	6. I am worried about the abuse of personal information	0.843	5.236			
	7. People around me may have a lower evaluation of me	0.789	6.382			
	8. The service failure made me feel bad	0.649	2.168			
Negative emotions	9. The service failure disappointed me	0.812	2.996	0.769	0.863	0.560
	10. Service recovery did not eliminate my complaint	0.636	3.289			
	11. I am angry about the OTA	0.783	6.032			
	12. I feel angry about the hotel	0.824	3.664			
	13. I regret choosing the OTA	0.666	3.072			
Corporate reputation	14. The OTA is trustworthy	0.905	6.137	0.857	0.902	0.650
	15. People around me have recommended the OTA to me	0.893	7.195			
	16. The OTA has a good reputation	0.782	5.396			
	17. There are few negative reports about the OTA	0.728	4.214			
	18. The OTA is a good partner in my life	0.673	2.797			
Service recovery satisfaction	19. I am satisfied with the timing of service recovery	0.677	2.825	0.741	0.839	0.513
	20. I am satisfied with the way of service recovery	0.779	2.821			
	21. Service recovery solves my problem	0.833	5.264			
	22. Service recovery meets my psychological expectation	0.626	6.259			
	23. I am satisfied with the service staff	0.643	3.998			
Customer loyalty	24. I will continue to purchase the services of the OTA	0.872	7.223	0.809	0.848	0.585
	25. I will recommend others to use the OTA's services	0.739	3.262			
	26. I will be a fan of the OTA	0.801	7.298			
	27. The OTA gives me a sense of belonging	0.627	4.465			

Based on reliability analysis, the validity of the questionnaire is analyzed. In terms of content validity, all items of the questionnaire refer to published authoritative journals, widely refer to the opinions of Chinese residents with the OTA service failure and recovery experience, and make adjustments combined with the OTA service recovery scenarios, which shows that the questionnaire items have good content validity. Convergence validity is shown in Table 2. The standardized load factors of all items are >0.5, the *T***-**value is greater than the threshold value of 1.96, the combined reliability (CR) values of the five variables are >0.7, and the average extraction variance (AVE) is >0.5, which meets the convergence validity test standard of Hair and Black (2006) and Wu (2010). Therefore, the questionnaire passed the convergence validity test.

The analysis of discriminant validity is shown in Table 3. The square root value of the AVE of the five variables is greater than the correlation coefficient between the variable and other variables. According to Wu (2010) criterion of discriminant validity, the questionnaire has good discriminant validity, and the discriminant validity has passed the test.

**Table 3:** Test for discriminant validity.

**Variables**	**1**	**2**	**3**	**4**	**5**
1. Perceived risk	0.773				
2. Negative emotions	0.597	0.776			
3. Corporate reputation	−0.194	−0.186	0.806		
4.Service recovery satisfaction	−0.358	−0.322	0.412	0.716	
5. Customer loyalty	−0.213	−0.303	0.314	0.589	0.765

*The value on the diagonal is the square root of AVE; and the other data are the correlation coefficients of the corresponding variables*.

In the test for construction validity, the measurement values of the research model fit are shown in Table 4: χ^2^/*df* = 2.752, CFI = 0.945, TLI = 0.942, SRMR = 0.039, RMSEA = 0.058. According to the good model standard of Wu (2010), χ^2^/*df* should be <5, CFI and TLI should be >0.9, SRMR should be <0.05, and RMSEA should be <0.1. Therefore, the above indicators of the research model have reached the standard of a good model, and the construction validity of the questionnaire has passed the test.

**Table 4:** Research model fit.

**Fit index**	**χ2**	***df***	**χ^2^/*df***	**CFI**	**TLI**	**SRMR**	**RMSEA**
Index value	308.215	112	2.752	0.945	0.942	0.039	0.058

### Test for Direct Impact Relationship

In this study, the SPSS22.0 software was used for multilevel regression analysis to test direct impact relationship and moderating role. In order to avoid multicollinearity, independent variables and regulatory variables were analyzed and treated centrally before multi-level regression analysis. The calculation results of the variance expansion factor (VIF) show that the VIF is between 2.072 and 3.382, which is lower than the empirical value of 10, indicating that there is no multicollinearity problem in the research model. The results of multilevel regression are shown in Table 5.

**Table 5:** Multilevel regression analysis.

**Variables**	**Negative emotions**		**Service recovery satisfaction**				**Customer loyalty**	
	**Model 1**	**Model 2**	**Model 3**	**Model 4**	**Model 5**	**Model 6**	**Model 7**	**Model 8**
Intercept	3.032^*^ (0.044)	2.839^**^ (0.032)	3.566^*^ (0.027)	2.681^*^ (0.089)	2.929^*^ (0.075)	3.528^*^ (0.109)	2.434^**^ (0.046)	3.633^**^ (0.113)
**Control variables**								
Gender	0.091	−0.022	0.091	0.014	0.011	0.122	0.023	0.044
Region	0.012	0.017	0.018	0.027	0.008	0.031	0.021	0.023
Age	−0.103^*^	−0.096	−0.073	−0.241	−0.111	−0.019	−0.099	−0.066
Education	0.028	0.024	−0.043^*^	0.208^*^	0.016	0.035^*^	0.031	0.011^*^
Occupation	0.018	0.013	−0.012	0.102	0.021	0.025	0.017	0.039^*^
**Independent variable**								
Perceived risk		0.697^**^		−0.589^*^		0.499^*^		
Negative emotions					−0.665^**^			
Service recovery satisfaction								0.708^**^
**Moderating variables**								
Corporate reputation						0.318^*^		
**Interaction item**								
Perceived risk × corporate reputation						0.217^*^		
*R^2^*	0.041	0.242	0.053	0.137	0.324	0.302	0.049	0.132
Δ*R^2^*		0.073		0.102	0.239	0.083		0.225
*F*	4.203^**^	2.807^**^	3.279^**^	1.986^*^	3.229^**^	2.906^**^	2.154^*^	2.652^**^

* *p <0.05*,

** *p <0.01*,

*** *p <0.001*.

This study constructs models 1 and 2 to test the direct relationship between perceived risk and negative emotions. As shown in Table 5, model 1 is the regression of negative emotions with control variables. The results show that there is a weak negative relationship between age and negative emotions, and that the relationship between gender, origin, education, and perceived risk is not statistically significant. Model 2 showed that perceived risk had a significant positive impact on negative emotions (β = 0.697, *P* < 0.01). At the same time, both models 1 and 2 passed the F-test, and the Δ*R*^2^ of model 2 was >0, indicating that the explanatory power of the model gradually increased. Therefore, hypothesis H1 passed the test.

The direct impact of perceived risk and negative emotion on service recovery satisfaction was tested by constructing models 3, 4, and 5. First, the control variables are included in model 3, which shows that there is a weak negative impact between education and perceived risk, and that the impact of gender, age, source region, and other control variables on perceived risk is not statistically significant. Second, according to the method of layer-by-layer inclusion, perceived risk is included in model 4. The results show that perceived risk has a significant negative impact on service recovery satisfaction (β = −0.589, *P* < 0.05), so research hypothesis H2 passes the test. On this basis, negative emotions were included in model 5. The results showed that negative emotions had a significant negative impact on service recovery satisfaction (β = −0.665, *P* < 0.01). The results supported hypothesis H3.

To test the relationship between service recovery satisfaction and customer loyalty, the study constructs models 7 and 8. First, the control variables are included in Model 7, which shows that the negative influence of education, age, occupation, and other control variables on customer loyalty is not statistically significant. Then, service recovery satisfaction is included in model 8. The results show that service recovery satisfaction has a significant impact on customer loyalty (β = 0.708, *P* < 0.01). The results support research hypothesis H5.

### Moderating Role Test

In this study, moderating role was also tested by multilevel regression analysis. As shown in Table 5, in model 6, the moderating role of corporate reputation between perceived risk and service recovery satisfaction is tested. From the multilevel regression results, it is found that the interaction coefficient between perceived risk and corporate reputation is positive and statistically significant (β = 0.217, *P* < 0.05), indicating that in the OTA service recovery, corporate reputation plays a positive moderating role in the relationship between perceived risk and service recovery satisfaction, and that research hypothesis H4 is supported.

**Figure 2:**
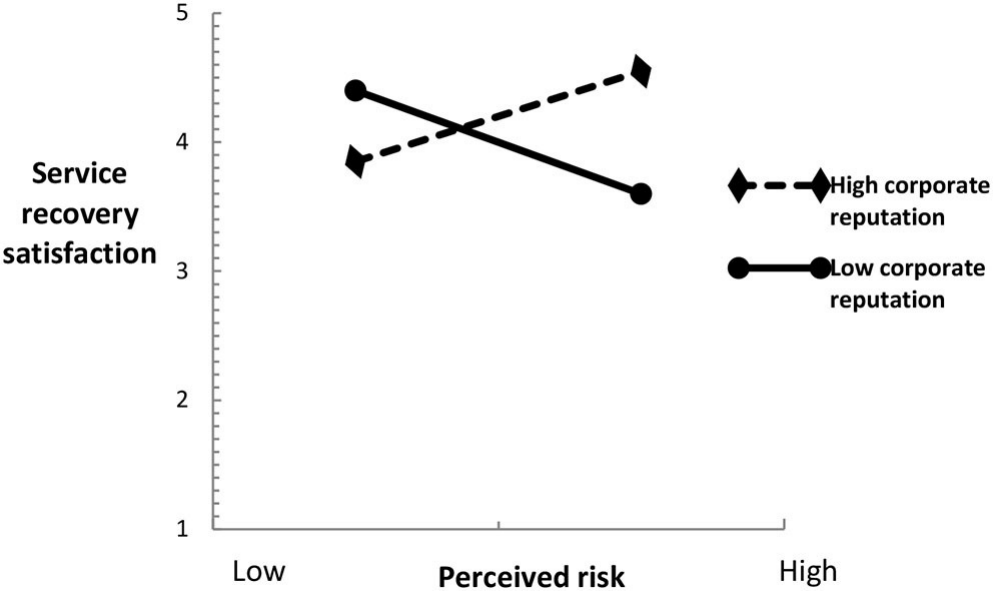
Moderating role chart.

To more clearly show the moderating role of corporate reputation between perceived risk and service recovery satisfaction, this study draws a moderating role diagram, as shown in Figure 2. The results of simple slope test show that perceived risk has a strong negative impact on service recovery satisfaction when corporate reputation is low (β = −0.728, *P* < 0.05). When corporate reputation is high, perceived risk has a weak negative impact on service recovery satisfaction (β = −0.316, *P* < 0.05). Therefore, when corporate reputation reduces the negative impact of perceived risk on service recovery satisfaction, it plays a positive moderating role. Research hypothesis H4 has been further verified.

### Mediating Effect Test

Because the mediating effect test in the study belongs to simple mediating effect test, and this kind of mediating test using Bootstrap method has obvious advantages, which will be more scientific and accurate than the causal stepwise regression method (Preacher et al., 2007). At the same time, the sampling performed in this study does not conform to the normal distribution, and the Bootstrap method does not need to assume normal distribution of sampling. It estimates the indirect effect and sampling distribution through repeated sampling, and estimates the confidence interval of indirect effect according to distribution characteristics (Preacher et al., 2007; Wu, 2010). Therefore, this study will use the Bootstrap method for mediating effect test.

The study constructs the competition model and chain mediating effect model to further confirm the existence of chain mediating effect. Table 6 shows the fit index of the competition model and the chain mediating effect model. According to Wu (2010), the standard of good model is that χ^2^/*df* is <5, CFI and TLI are >0.9, SRMR is <0.05, and RMSEA is <0.1. As shown in Table 6, the χ^2^/*df* value of the competition model is 6.363, and other fitting indexes do not meet the good requirements, indicating that the fitting degree of the competition model is poor. The χ^2^/*df* , CFI, TLI, SRMR, and RMSEA fit indexes of the chain mediating effect model all meet the requirements of a good model and are significantly better than the competition model parameters. Therefore, the chain mediating effect does exist.

**Table 6:** Test of competition model of mediating effect.

**Model**	**χ^2^**	***df***	**χ^2^/*df***	**CFI**	**TLI**	**SRMR**	**RMSEA**
Chain mediating effect	57.793	21	2.752	0.934	0.925	0.037	0.054
Competition model	178.176	28	6.363	0.711	0.802	0.074	0.123

The mediating effect test is shown in Table 7. The indirect effect value of mediating path “perceived risk → service recovery satisfaction → customer loyalty” is 0.276, accounting for 46.7% of the total effect. It shows that service recovery satisfaction plays a mediating effect between perceived risk and customer loyalty. Research hypothesis H6 passes the test. The indirect effect value of mediating path “perceived risk → negative emotion → service recovery satisfaction → customer loyalty” is 0.132, accounting for 22.33% of the total effect, which indicates that negative emotion and service recovery satisfaction play a mediating effect between perceived risk and customer loyalty. Research hypothesis H7 passes the test. As shown in Table 7, the confidence intervals of the total mediating effect and the two mediating effects do not contain a value of 0, and are statistically significant. The total mediating effect was 0.408, accounting for 69.03% of the total effect.

**Table 7:** Test results of mediating effect.

**Mediating effect path**	**Indirect effect value**	**Standard error**	**Upper limit**	**Lower limit**	**Effect proportion**
1. Perceived risk → service recovery satisfaction → customer loyalty	0.276	0.019	0.164	0.468	46.7%
2. Perceived risk → negative emotion → service recovery satisfaction → customer loyalty	0.132	0.008	0.123	0.391	22.33%
4. Total mediating effect	0.408	0.027	0.327	0.702	69.03%
5. Total effect	0.591	0.036	0.214	0.575	100%

## Conclusion and Discussion

### Conclusions and Theoretical Contributions

First, the results show that: in the OTA service recovery scenario, the perceived risk of customers has a significant positive impact on negative emotions, and that the perceived risk and negative emotions of customers have a significant negative impact on service recovery satisfaction. Therefore, the above results support hypotheses H1, H2, and H3. H1 is supported, which shows that in the OTA service recovery scenario, the greater the perceived risk, the greater the negative emotion. This research conclusion extends the research scenario to that of OTA service recovery, which is an extension and enrichment of the conclusions of Lee and Jung (2015) and Mi et al. (2019). This study verifies hypothesis H2, which confirms and extends the conclusions of Hsieh and Tsao (2014), Chang and Hsiao (2008), and Scridon et al. (2020) that perceived risk has a negative impact on customer satisfaction and service recovery satisfaction. This study confirms that the perceived risk of customers will have a negative impact on service recovery satisfaction in the OTA service recovery scenario. At the same time, this study also verifies research hypothesis H3, which indicates that negative emotions of customers will also have a negative impact on service recovery satisfaction. In previous studies, Du and Fan (2007) and Yoon and Lee (2014) have suggested that negative emotions will negatively impact customer satisfaction and service recovery satisfaction. The conclusion of this study is an extension of previous studies. In short, through empirical research, research hypotheses H1, H2, and H3 are supported, which expand the connotation and applicable scenarios of the relationship between perceived risk, negative emotions, and service recovery satisfaction, and expand the connotation and applicable scenarios of the relationship between negative emotions and service recovery satisfaction.

Second, according to the results of this study, corporate reputation plays a positive moderating role in the relationship between perceived risk and service recovery satisfaction. Therefore, hypothesis H4 is supported. In the OTA service recovery scenario, with the improvement in OTA corporate reputation, service recovery satisfaction will increase faster. The corporate reputation of an OTA will positively moderate the relationship between perceived risk and service recovery satisfaction, that is, reduce the negative impact of perceived risk on service recovery satisfaction. The existing literature suggests that the corporate reputation of an online platform can maintain long-term psychological contract relationship, create better relationship, and improve demand (Li, 2014). However, there is insufficient research on the moderating role of corporate reputation between perceived risk and service recovery satisfaction under the OTA service recovery scenario. This study verifies that the corporate reputation of an OTA can positively moderate the negative impact of perceived risk on service recovery satisfaction, which makes up for the lack of current research. This study provides a theoretical and empirical explanation to further explore the regulatory mechanism of the corporate reputation of OTAs and is helpful for the majority of service enterprises, especially OTAs, in improving their reputation or re-evaluating their image.

Third, in the OTA service recovery scenario, service recovery satisfaction has a positive impact on customer loyalty, and research hypothesis H5 has passed the hypothesis test. Negative emotions and service recovery satisfaction play a mediating effect between perceived risk and customer loyalty. Hypothesis H6 passes the hypothesis test. The research also shows that service recovery satisfaction also plays a mediating role between perceived risk and customer loyalty, and that research hypothesis H7 also passes the hypothesis test. The conclusion that service recovery satisfaction has a positive impact on customer loyalty further confirms the research results of Yu et al. (2008), extends the research scenario to that of OTA service recovery, and expands the applicable scenarios and research connotation of service recovery satisfaction and customer loyalty. However, in the OTA service recovery scenario, there are few research studies on the mediating effects of negative emotions and service recovery satisfaction between perceived risk and customer loyalty. This study makes a breakthrough and innovation in this aspect.

### Practical Applications

First, after service failure, OTAs and their service providers should manage the negative emotions of customers and optimize service recovery strategies. Because of service failure, the perceived risk of customers to OTAs and service providers will increase, and negative emotions, such as complaints, disappointment, and helplessness will follow. This study shows that perceived risk and negative emotions have a negative impact on service recovery satisfaction. Therefore, OTAs and service providers should manage and guide emotions of customers through the negative emotion management scheme designed by psychological and service management experts, enriching service recovery means, combining material compensation with spiritual compensation, timely carrying out personalized service recovery, taking interests of customers into consideration, and improving service recovery satisfaction.

Second, OTAs should strive to maintain and enhance their corporate reputation and do everything possible for the sake of customers to improve corporate reputation. The results show that corporate reputation reduces the negative impact of perceived risk on service recovery satisfaction. If an OTA has a good corporate reputation, in the process of service failure and service recovery, the negative effect of perceived risk on service recovery satisfaction will be reduced, and it will become the “protector” of enterprises to resist risks. However, for an OTA, a good corporate reputation cannot be formed overnight. It needs a long-term accumulation of OTA, which is the embodiment of corporate image. In the era of the Internet, OTAs should be honest, put an end to cheating customers, do everything possible for customers, and establish a good brand image. At the same time, OTAs need to carry out public relations and promotion work, and use news media, hot events, and public welfare activities to improve corporate reputation.

Third, OTAs should improve customer loyalty through effective service recovery. After OTA service failure, customers are very disappointed with OTAs, but they can reverse their attitude through service recovery. From the perspective of this study, reducing the perceived risk and negative emotions of customers is only an important incentive to enhance customer loyalty, not a direct factor. Therefore, in OTA service recovery, we should pay attention to the formation of service recovery satisfaction. OTAs should take scientific and effective service recovery measures to reduce perceived risks and negative emotions of customers, scientifically guide customers, be good at approaching customers, resolve negative emotions of customers, solve concerns of customers about money and time, and improve service recovery satisfaction. On this basis, customers will continue to patronize the OTA and recommend it to their relatives and friends, so as to form customer loyalty and become loyal customers of the OTA.

### Research Limitations and Prospects

First, future research needs to extend the research framework. The research framework of this study does not involve customer trust, consumer value, service quality, consumer perception, and other aspects, but in the process of service recovery, the above four variables may have an important impact on the effect of service recovery. In future research, we will extend the research framework by including consumer trust, consumer value, service quality, consumer perception, and other variables in order to better explore the impact mechanism of OTA service recovery effect. For example, we will study the mediating role of consumer trust between service recovery satisfaction and customer loyalty, or the mediating role of consumer trust between consumer forgiveness and customer loyalty. The purpose of the study is to examine the impact of consumer values on consumer emotion and service recovery satisfaction, find out the differences in the impact of consumers with different values on related variables, explore the impact of service quality on the relationship between negative emotions and service recovery satisfaction, and research on the relationship between consumer perception and emotion type, service quality and service recovery satisfaction, etc. In addition, in future research, this study can also go beyond the current research framework to explore if consumer distrust will lead to disapproval of OTAs and cause damage to the image and reputation of OTAs. These research ideas will expand the vision of OTA service recovery research and enrich the accumulation of service recovery theory.

Second, future research needs to optimize research methods. Although the scenario experiment is suitable for collecting customer data and has high internal validity, these data are all from the subjective evaluation of customers and have certain limitations. In future research, we will collect objective data of customers at the same time. Objective data mainly refer to the data generated by customers using OTA platforms, and they include the login time and times of customers on OTA platforms, length of stay of customers on OTA platforms, proportion of customers choosing various types of hotels on OTA platforms, and so on. Obtaining objective data will make up for the deficiency in scenario experiment, because subjective evaluation results of customers often have certain deviation. Therefore, the combination of objective and subjective data will be the direction of future research.

Third, future research needs to expand the source of samples. The samples in the study come from Guilin, Nanning, and Liuzhou in China, but there are no samples from other countries, especially from Southeast Asia. In future research, we will expand the scope of sample sources, investigate samples from many countries, expand sample size, and improve representativeness of samples. At the same time, adaptive joint analysis (ACA) embedded in polyhedral methods will be used to reduce the complexity of the questionnaire.

## Data Availability Statement

The raw data supporting the conclusions of this article will be made available by the authors, without undue reservation.

## Ethics Statement

This research is approved by the Ethics Committee of Guilin University of technology, which belongs to Guilin University of technology. The patients/participants provided their written informed consent to participate in this study.

## Author Contributions

JW independently completed the paper, carried out literature analysis, scenario experiment and data analysis, and demonstrated the research hypothesis, as well as other work in the paper.

## Conflict of Interest

The author declares that the research was conducted in the absence of any commercial or financial relationships that could be construed as a potential conflict of interest.
